# Preparing Osteopathic Students for the Single Graduate Medical Education Accreditation System: Evaluating Factors for Match Success in Emergency Medicine

**DOI:** 10.5811/westjem.2018.6.37922

**Published:** 2018-07-26

**Authors:** Megan Stobart-Gallagher, Alanna O’Connell

**Affiliations:** Albert Einstein Medical Center, Department of Emergency Medicine, Philadelphia, Pennsylvania

## Abstract

**Introduction:**

With the development of and progression toward a single graduate medical education accreditation system combining the current Accreditation Council for Graduate Medical Education (ACGME) and American Osteopathic Association (AOA) residency programs, the total number of students competing for the same postgraduate training spots will continue to rise. Given this increasing competition for emergency medicine (EM) residency positions, understanding factors that contribute to match success is important to ensure a successful match for osteopathic medical students.

**Methods:**

Our anonymous survey to evaluate factors that led to a successful match was sent out to residents in current ACGME-, AOA-, and dually-accredited programs via the AOA program director listserv and the Council of Residency Directors (CORD) e-mail listserv in 2017.

**Results:**

We had 218 responses. Responses showed that osteopathic graduates had less affiliation with EM residencies, their home institutions provided less information regarding standardized letters of evaluations (SLOE), and that successful osteopathic graduates seemed to learn about them while on EM elective rotations. These students also had less direct EM mentorship and were generally unsatisfied with the level of mentorship available. Osteopathic graduates in current ACGME programs were also more likely to have taken the United States Medical Licensing Examination compared to their AOA resident counterparts.

**Conclusion:**

Osteopathic medical schools can improve their graduates’ chances of successfully matching in EM by establishing mentorship programs and educating their students early about SLOEs.

## INTRODUCTION

The second semester of the fourth year of medical school is generally regarded as the least stressful in a medical school, with one notable exception: the match. Students hoping to obtain a residency position in emergency medicine (EM) face increasingly steep competition. The 2016 data from the National Resident Matching Program (NRMP) (allopathic) match showed 2,703 applicants for a total of 2,047 EM positions, with only four unfilled spots after the main match.^1^ Of the 264 osteopathic applicants participating in this match, 60 went without a successful EM match. In the National Matching Services (NMS) (osteopathic) match, 310 positions were available in EM with again only four unfilled spots after the main match.^2^ Osteopathic graduates have historically made up a small percentage of the total participants in the NRMP match – 8.4%^3^ in 2017. As we move toward a single graduate medical education (GME) accreditation system, the number of osteopathic students competing with allopathic students will continue to rise. The failure in the past year of some osteopathic students to match was likely multifactorial. Given the increasing competition for EM residency positions, understanding factors that contribute to match success is becoming increasingly important to ensure that strong osteopathic candidates are not overlooked.

Our objective was to query active EM residents and, by retrospectively reviewing the steps they took, to understand any potential limitations that current osteopathic students may face to achieve a successful match. We aimed to identify these limitations well in advance of the merger in order to guide students prospectively as they apply for EM residency positions. We hypothesized that osteopathic medical students are at a particular disadvantage compared with their allopathic peers, especially in terms of EM-specific mentorship at their respective undergraduate institutions.

## METHODS

With approval from our institution’s institutional review board, we created an anonymous retrospective survey using Google Forms and distributed it to active, consenting residents in current Accreditation Council for Graduate Medical Education (ACGME)-, American Osteopathic Association (AOA)-, and dually-accredited programs, via the AOA program director listserv and the Council of Emergency Medicine Residency Directors Advances in Education Research and Innovations (CORD) listserv in the spring of 2017, just after the match.

**Figure 1:**
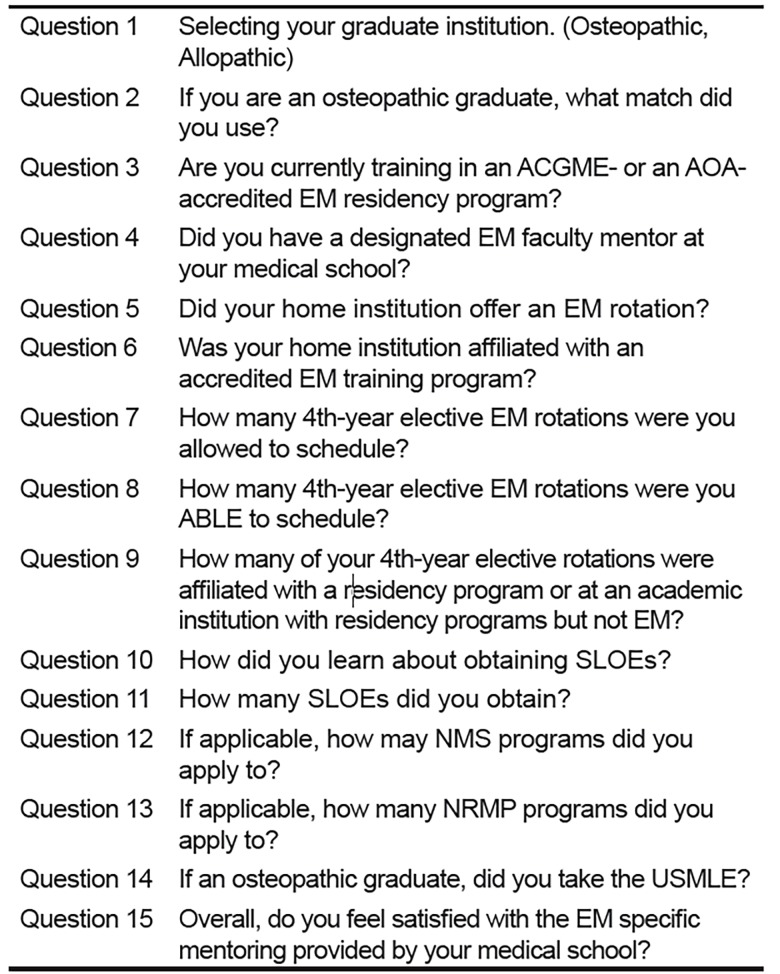
Survey questions distributed to residents through Google forms. *ACGME*, Accreditation Council for Graduate Medical Education; *AOA*, American Osteopathic Association; *EM*, emergency medicine; *SLOE*, standardized letter of evaluations; *NMS*, National Matching Services; *NRMP*, National Resident Matching Program; *USMLE*, United States Medical Licensing Examination.

We collected survey results using Google Forms and analyzed them with Microsoft Excel (Redmond, WA). We received a total of 218 responses, which we sorted into two groups to allow for comparison: respondents who graduated from an allopathic medical school and respondents who graduated from an osteopathic medical school. We did not differentiate responses based on postgraduate-year level. Survey questions highlighted multiple aspects of the match process including board scores, standardized letters of evaluation (SLOE) and mentorship, among others (Figure 1).

We tallied and calculated responses as a percentage of that group’s (osteopathic/allopathic) responders. Percentages were rounded to nearest percentage for better visualization and comparison.

## RESULTS

Of the 218 responses to our survey, 119 (54%) were from residents who graduated from an allopathic medical school and 99 (45%) from residents who graduated from an osteopathic medical school. The majority of responses, 64%, came from residents currently training at an ACGME program, 28% were at a dually- accredited program, and 7% were at an AOA program.

Of the 99 osteopathic resident responses, only 27% reported a medical school affiliation with an EM residency as compared to 73% noted by the allopathic graduates. Most allopathic graduates had an EM rotation offered by their home institution (80%), as compared to osteopathic graduates who reported only 35% of them came from programs that offered an EM rotation at their home institution. There was less of a contrast between groups when comparing numbers of total EM rotation opportunities they were allowed to schedule. Allopathic graduates reported that 44% were allowed to schedule >3 rotations in EM. Osteopathic graduates reported 56% were allowed to schedule >3 rotations in EM.

**Population Health Research Capsule**

What do we already know about this issue?

Applying for residency has become increasingly competitive. Traditionally osteopathic medical students have made up a small percentage of participants in the National Resident Matching Program. As we move to a single graduate medical education accreditation system more osteopathic students will be compared to their allopathic counterparts.

What was the research question?

What potential limitations may osteopathic students face to achieve a successful match?

What was the major finding of the study?

Osteopathic graduates do not have the same level of pre-residency resources as allopathic students, particularly with fewer affiliated EM residency programs, and fewer mentorship opportunities.

How does this improve population health?

These data demonstrate that osteopathic medical schools can make their students more competitive for EM residency positions to ensure that no qualified applicant is overlooked in the future.

**Figure 2:**
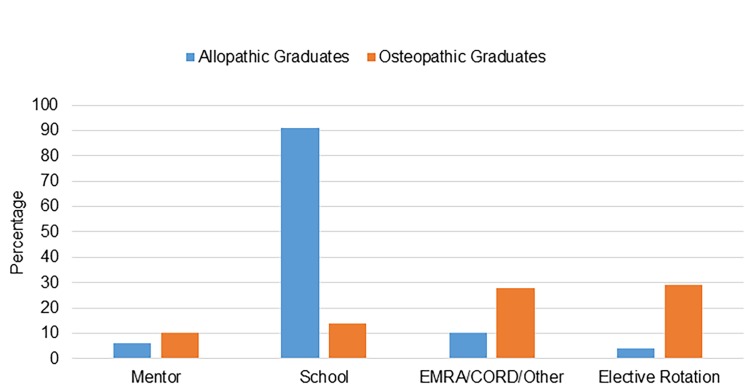
How survey respondents learned about the standardized letter of evaluation. *EMRA*, Emergency Medicine Residents’ Association; *CORD*, Council of Emergency Medicine Residency Directors Advances in Education Research and Innovations.

The responses were more varied regarding how students learned about SLOEs. The four most common responses were mentors, medical schools, sites such as the EM Residents’ Association (EMRA)/CORD, and elective rotations. The osteopathic and allopathic groups again had differing responses to this question. Allopathic graduates most commonly learned about SLOEs from their medical schools (91%), followed by EMRA/CORD (10%), mentors (6%), and electives (4%). Osteopathic graduates more commonly learned about SLOEs while on their EM-elective rotation (29%), through EMRA/CORD (28%), school (14%), and mentors (10%) (Figure 2).

**Figure 3:**
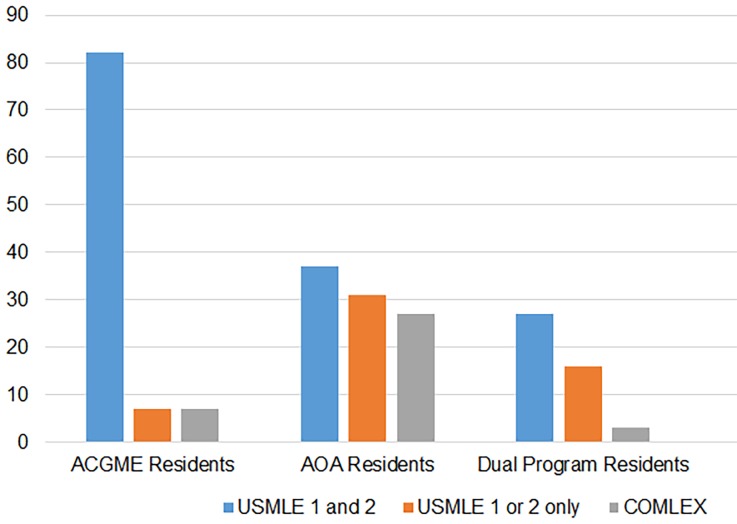
Board exams taken by allopathic and osteopathic residents in ACGME-, AOA-, and dually-accredited programs.

For osteopathic graduates in ACGME programs, 82% had taken the United States Medical Licensing Examination (USMLE) Steps 1 and 2 (7% did not take USMLE exams, and 7% took either Step 1 or Step 2 only). In AOA programs, 37% took USMLE Steps 1 and 2 (25% did not take the USMLE, and 31% took either Step 1 or Step 2); and in dual programs 27% took USMLE Steps 1 and 2. Of note, 57% in dual programs did not take the USMLE at all (16% took either Step 1 or step 2) (Figure 3).

**Figure 4:**
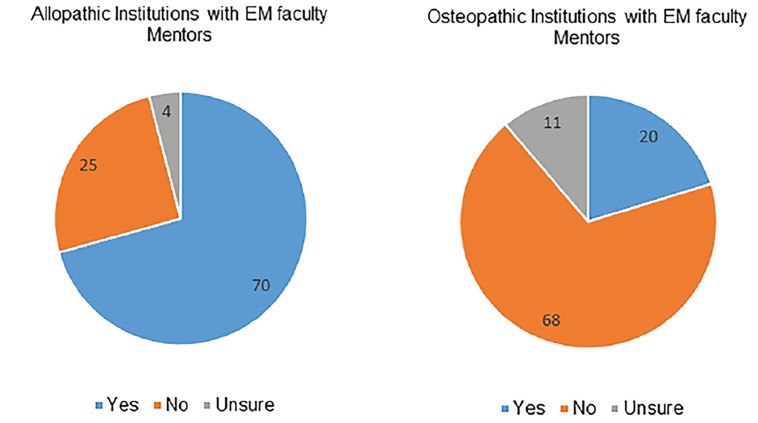
Availability of mentors at allopathic and osteopathic medical schools. *EM*, emergency medicine.

**Figure 5:**
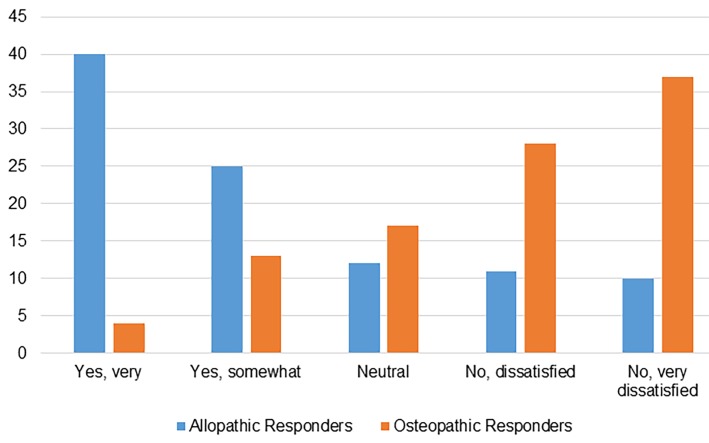
Satisfaction with available mentorship as reported by osteopathic and allopathic graduates.

Regarding EM-specific mentoring, allopathic graduates predominantly had structured mentoring support; 70% of allopathic responders reported that their home institution had an EM faculty mentor, 30% did not have a mentor, and 4% were unsure. This was in stark contrast to osteopathic responders who reported that only 20% had an EM faculty mentor at their home institution, while 68% reported no mentor and 11% were unsure (Figure 4). Levels of satisfaction with available mentoring were also different between the two groups. Of the allopathic graduates, 65% reported they were overall satisfied, 12% were neutral, and 21% were dissatisfied. Of the osteopathic graduates, 17% were satisfied, 17% were neutral, and 65% were dissatisfied (Figure 5).

## DISCUSSION

In reviewing the survey data, we found that our responses were almost evenly divided between the doctor of osteopathic medicine (DO) and doctor of medicine (MD) groups. Most of the graduates (DO and MD) who responded are currently training at ACGME programs, thus representing the population we most wanted to study. In the responses we received, the general theme appeared to be that osteopathic graduates do not have the same level of pre-residency resources or support as their allopathic colleagues. This is likely part of a multifactorial problem and a product of the different environments between osteopathic and allopathic medical schools; however, our study did suggest some areas where improvement could be made.

Because osteopathic medical schools are typically not affiliated with a major academic institution, it was not surprising that the majority of osteopathic graduates did not have an affiliated EM residency with their school. Most medical schools regardless of type appear to be supportive in allowing their students to participate in EM electives. The majority of survey responders stated that their school allows >3 electives to be scheduled.

Students in ACGME-accredited programs primarily took USMLE Steps 1 and 2 based on survey responses. This could be multifactorial and our assumption would be that more students may have taken this exam due to a perceived preference by residency programs, or perhaps osteopathic students were attempting to appear more competitive by taking the additional exam. Graduates currently in dually-accredited programs seem to match well without taking the USMLE; this was also likely multifactorial. Again, we could assume that dual programs are likely more familiar with the Comprehensive Osteopathic Medical Licensing Examination (COMLEX), or perhaps these students had higher COMLEX scores and did not feel the need to take an additional exam. Further studies could be directed at ascertaining the reasons for this.

Obtaining SLOEs is something most allopathic students learn about from their medical schools. The majority reported that was where they learned about SLOEs, with the second highest number stating they learned from a source such as CORD/EMRA. Osteopathic students had a wider variety of responses but, notably, far fewer had learned about this vital part of the application process from their medical school. They seemed to learn about the necessity of getting SLOEs during their elective rotations rather than beforehand, which could have led to obtaining letters late in the application season.

The majority of allopathic responders reported having mentoring available to them directly from their medical school, as opposed to the osteopathic responders who reported the majority did not have EM-specific mentorship available to them. Osteopathic responders appeared to be dissatisfied overall with the level of mentorship available to them, as demonstrated by their responses.

## LIMITATIONS

One limitation in our study was the low response rate to the survey. Based on an estimation of the current number of U.S. medical school graduates in EM training, excluding international graduates, our response rate was approximately 5%. While this low response rate likely limited the major conclusions that we could draw, the survey results do suggest an overall trend. Perhaps future studies could draw an improved response rate by direct communication with programs and residents. We did, however, have a nearly even number of responses between DO and MD residents and thus believe we obtained a representative sample of the population we were studying.

## CONCLUSION

Osteopathic medical students face a disadvantage in the EM match in multiple areas. Fewer osteopathic graduates came from schools with EM residency affiliations or learned about SLOEs from their medical schools. They reported having less mentorship during their undergraduate studies and overall felt dissatisfied with the level of mentorship available to them. Our study suggests that osteopathic medical schools could improve their graduates’ chances of successfully matching in EM by establishing mentorship programs and educating their students early about SLOEs. Obtaining affiliations with EM residency programs would be beneficial as well. As we move to a single match by 2020 under the single GME accreditation system, encouraging students to take the USMLE could also prove advantageous, given that the majority of osteopathic graduates at ACGME-accredited programs had taken that exam.
